# Presence of Inflammatory Group I and III Innate Lymphoid Cells in the Colon of Simian Immunodeficiency Virus-Infected Rhesus Macaques

**DOI:** 10.1128/JVI.01914-19

**Published:** 2020-04-16

**Authors:** Andrew Cogswell, Natasha Ferguson, Edward Barker

**Affiliations:** a Department of Microbial Pathogens and Immunity, Rush University Medical Center, Chicago, Illinois, USA; Emory University

**Keywords:** SIV, colon, cytokines, gut inflammation, innate lymphoid cells, rhesus macaque

## Abstract

Chronic, low-grade, systemic, and mucosal inflammation correlates with increased morbidity and poor clinical outcomes among patients living with human immunodeficiency virus (HIV). These long-term complications are linked to the disruption of gastrointestinal (GI) tract epithelial barrier integrity and subsequent microbial translocation. However, the mechanisms responsible for these downstream effects of infection are unknown. Here, we demonstrate that during the disruption of the GI tract and increased microbial translocation, we find inflammatory cytokines (e.g., interferon gamma [IFN-γ] and tumor necrosis factor alpha [TNF-α]) produced by innate lymphoid cells (ILCs) located in the colon secondary to simian immunodeficiency virus (SIV) infection. To do this, we used viably cryopreserved colon cells from SIV-infected and uninfected rhesus macaque monkeys and determined the make-up of the ILC subpopulations and the cytokines they expressed constitutively. Our studies revealed that the interleukin-22 (IL-22)/IL-17-producing ILCS was not altered during SIV infection. However, the percentage of IFN-γ^+^ ILCs in infected colons was 5- to 10-fold higher than that in uninfected colons. ILCs from infected tissue that produced IFN-γ also expressed TNF-α and IL-22. The coexpression of inflammatory cytokines with IL-22 is linked to the ability of ILCs to coexpress T-bet and RORγT/Ahr. The expression of IFN-γ/TNF-α by ILCs and NK cells combined likely triggers a pathway that contributes to chronic mucosal inflammation, GI barrier breakdown, and microbial translocation within the context of SIV/HIV infection.

**IMPORTANCE** There is a slow yet significant uptick in systemic inflammation secondary to HIV infection that has long-term consequences for the infected host. The systemic inflammation most likely occurs as a consequence of the disruption of the gut epithelial barrier, leading to the translocation of gut microbial products. This disruption may result from mucosal inflammation. Here, we show in an animal model of HIV that chronic SIV-infected gut contains innate lymphoid cells producing inflammatory cytokines.

## Abstract (precis)

There is a slow yet significant uptick in systemic inflammation secondary to HIV infection that has long-term consequences for the infected host. The systemic inflammation most likely occurs as a consequence of the disruption of the gut epithelial barrier, leading to the translocation of gut microbial products. This disruption may result from mucosal inflammation. Here, we show in an animal model of HIV that chronic SIV-infected gut contains innate lymphoid cells producing inflammatory cytokines.

## INTRODUCTION

During human immunodeficiency virus/simian immunodeficiency virus (HIV/SIV) infection, there is a rapid and massive depletion of CD4^+^ T cells within the mucosa of the gastrointestinal (GI) tract (1, 2), as well as the development of a compromised epithelial barrier, that leads microbial products to move from the lumen of the gut to the mucosa and, eventually, the periphery (3, 4). Microbial translocation (MT) is accompanied by prolonged mucosal and systemic immune activation, which, in turn, correlates with increased viremia and disease progression of infected patients (5, 6). Increased intestinal permeability likely promotes chronic systemic inflammation (7–9). Although there have not been reports of intestinal barrier restoration and consequent reductions of systemic inflammation in HIV-infected patients, recent data demonstrate that the increased permeability of intestinal epithelial tight junctions (TJs) is necessary for persistence (10). Several possible mechanisms drive epithelial barrier breakdown during SIV/HIV infection (5, 6), for example, reports suggest that the proinflammatory cytokines interferon gamma (IFN-γ) and tumor necrosis factor alpha (TNF-α) trigger the breakdown of tight junctions between GI tract epithelial cells (11–13). We previously reported that innate lymphoid cells (ILCs) are a source of IFN-γ in the colons of individuals with chronic untreated HIV-1 infection and were associated with gut dysbiosis and mucosal immune activation (14). These findings suggest a link between inflammatory ILCs and HIV mucosal pathogenesis. However, studies definitively identifying the mechanisms leading to the increased number of inflammatory ILCs in the GI tract during HIV infection are lacking.

ILCs reside within mucosal tissues and are the innate counterpart of effector T cells. These cells provide an immediate source of inflammatory mediators during microbial infection of mucosal tissues, thereby aiding in pathogen containment and clearance (15). ILCs are also involved in the homeostatic maintenance of the mucosal tissue (15). In contrast to effector T cells, ILCs do not express antigen-specific receptors or undergo clonal selection (16). Instead, ILCs react promptly to factors (e.g., cytokines) from the local mucosal environment (16). In this way, ILCs respond rapidly to many different pathogens that encroach upon mucosal tissues and, in turn, prevent pathogens from spreading and causing tissue damage. Since there are no specific markers that define ILCs and their subsets, experts have adopted guidelines describing ILC phenotype, function, and transcription factor expression (17). These guidelines identified three groups of ILCs. Group 1 ILCs, which include natural killer cells (NKs) and ILC1s, secrete inflammatory cytokines (e.g., IFN-γ and TNF-α) in response to proinflammatory signals, such as interleukin-12 (IL-12)/IL-18, and depend on the expression of transcription factor T-box 21 (T-bet) for their function (18). Group 2 ILCs require retinoic acid receptor-related orphan nuclear receptor alpha (RORα) and GATA binding protein 3 (GATA-3) transcription factors and secrete type 2 cytokines in response to “danger signals” from various cells, like epithelial cells (19, 20). Group 3 ILCs utilize ROR-γT for their effector function and secrete cytokines, such as IL-22, in response to IL-23 and IL-1β (21). Notably, IL-22 is vital in maintaining the health and integrity of the epithelial cell layer throughout the GI tract by various mechanisms (22–25). Recently, a unique group of ILCs with regulatory function (i.e., ILCreg) was identified (26). This ILC group has a phenotype and function similar to those of Treg, except they are antigen independent.

The fates and functions of ILCs within the GI tract in the context of HIV/SIV infection remain unclear. Some have reported a loss of colonic ILC3s secreting IL-22/IL-17 during chronic HIV infection in the absence of combined antiretroviral therapy (cART) (27, 28). However, we (14) and others (28–30) observed no reduction of ILCs in the colons of chronically HIV-infected patients in either the absence or the presence of cART. A more recent study that utilized a more extensive panel of markers to identify ILC3s found significantly higher frequencies of ILC3s with the potential to express IL-22, despite an overall reduction in cell numbers in the colon of HIV-infected patients undergoing cART compared to that of healthy controls (31). Interestingly, this study highlights the possibility that the frequency of ILC3s during HIV infection are site specific, as they found no significant differences in the ILC3 frequency in the ileum or duodenum of the same study participants (31). Studies involving nonhuman primates are similarly varied. Some studies have reported that during SIV infection in rhesus macaques, levels of colonic ILC3s, which have the potential to secrete IL-22/IL-17, were decreased relative to those of ILC3s from uninfected controls (32–35). Another study indicates that the frequency of ILC3s was lower in the jejunum but not in the colon of chronic SIV-infected animals (36).

Although, in principle, ILCs are categorized into three groups, in practice they are incredibly plastic, and their function and phenotype are modulated by environmental cues (37–39). Plasticity may be a factor contributing to the differing reports of frequency and function during disease states. For example, clones of ILC3 cells lose CD117 (i.e., c-Kit) but retain NKp44 surface expression and take on ILC1 function (e.g., IFN-γ expression) as a consequence of exposure to IL-12 (38, 39). In contrast, the same ILC3 clones retain c-Kit and IL-22 expression when exposed to IL-23 and IL-1β (39). ILC1 clones exposed to IL-23 and IL-1β secrete IL-22 and gain c-Kit and NKp44 surface expression (39). Similarly, ILC2s become functionally and phenotypically like ILC1s when exposed to IL-12 or ILC3s when exposed to IL-1β and IL-23 (40).

We have previously shown that within the context of chronic, untreated HIV-1 infection, there is an increase in frequencies of NKp44^+^ ILCs that potentially express IFN-γ (40). In uninfected study participants, these cells had the potential to express IL-22 but not IFN-γ when exposed to exogenous stimuli (40). Similarly, another study reported a higher frequency of colonic NKp44^+^ ILCs, potentially expressing IFN-γ in chronic SIV infection (32). However, in this study, it was unclear if the cells were ILC3s that had switched to an inflammatory profile, or, instead, the changes reflected an influx of ILC1s/NKs, given that NKp44 was shown to be expressed by both ILC3s and ILC1s (38, 39). These studies led us to hypothesize that the plastic nature of mucosal ILCs, generated during the diseased state of HIV/SIV, makes it possible for IL-17/IL-22 and IFN-γ/TNF-α to be expressed by both ILC3s and ILC1s. Thus, we performed the current *ex vivo* study to determine the frequency and number of ILC3s, ILC1s, and NKs that constitutively secrete IL-22, IL-17, and IFN-γ in the lamina propria of the colon of SIV-infected rhesus macaques relative to uninfected control animals.

## RESULTS

### Chronic SIV infection does not alter the frequency or the combined total number of ILC3s, ILC1s, and NKs within the colon.

**FIG 1:**
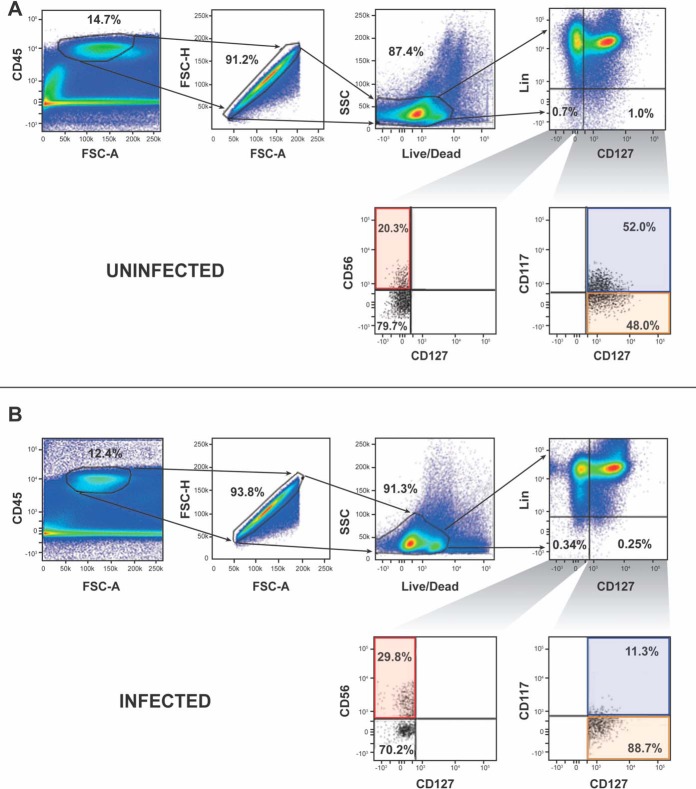

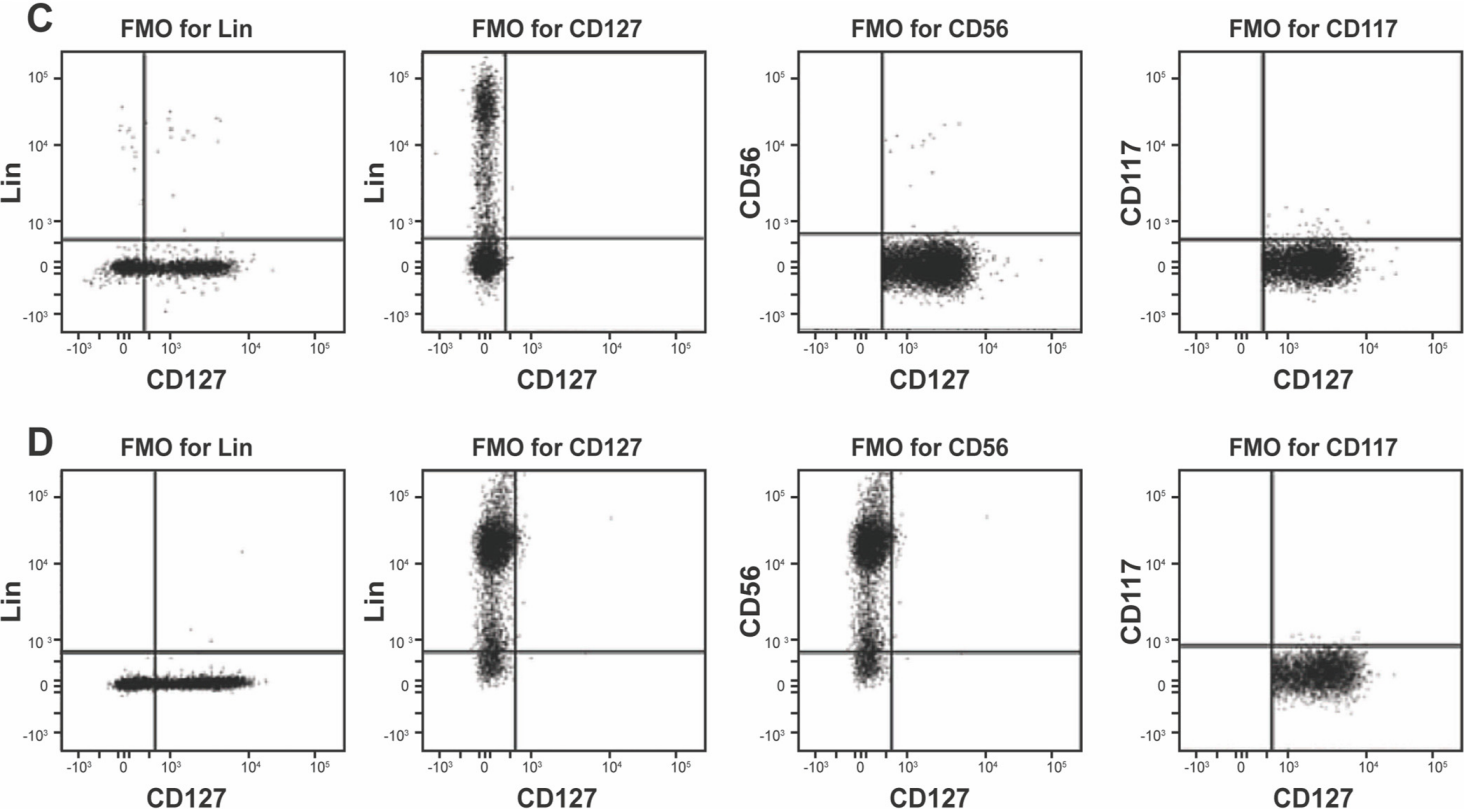
Gating strategy for assessing colonic ILCs and NKs of uninfected and SIV-infected rhesus macaques. (A and B) The gating strategy for defining ILCs and NKs from uninfected (A) and infected (B) colons involved selecting CD45-expressing, single cells that were viable. CD45^+^ viable single cells, which were within the lineage (Lin; CD3, CD11c, CD20, CD34, CD123, CD303, FCεR1) gate, were excluded from our analysis. Cells outside the Lin gate were further evaluated for cells expressing CD127. Cells lacking CD127 but possessing CD56 were considered CD56^+^ NKs. The CD127-expressing cells were evaluated for CD117 expression. Cells within the CD127 gate, which were CD117^+^, were ILC3s and ILC2s, whereas the cells lacking CD117 were ILC1s. FSC, forward scatter; SSC, side scatter. Fluorescent minus one (FMO) controls were used to set gates for determining the frequency of ILCs and NKs from uninfected (C) and SIV-infected (D) colons.

**FIG 2:**
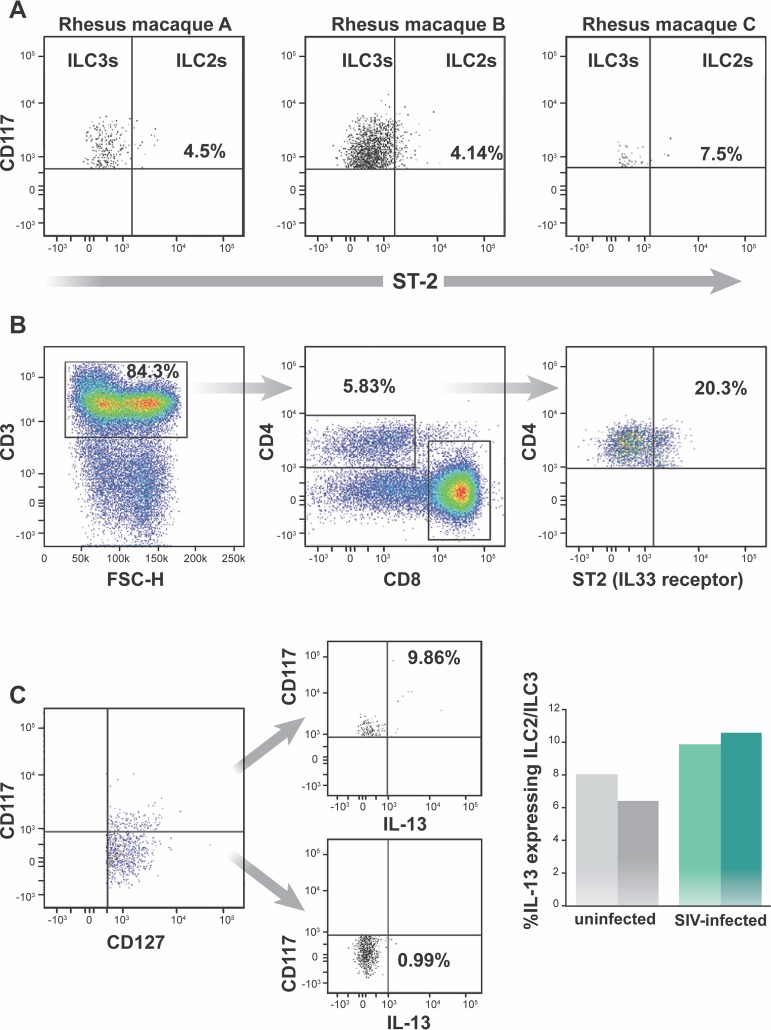
Frequency of ILC2s among CD45^+^ Lin^−^ CD127^+^ CD117^+^ cells in the colons of uninfected and SIV-infected rhesus macaques. (A) ILC2s were detected in the colons of three SIV-infected rhesus macaques. Fluorescent minus one (FMO) controls were used to set the gates to identify ST2 cells. (B) To confirm ST2 staining, CD45^+^ CD3^+^ CD4^+^ cells were stained for ST2 in SIV-infected rhesus macaques. This is representative of three experiments. (C) IL-13 production by CD45^+^ Lin^−^ CD127^+^ CD117^+^ cells in the colons of two SIV-infected and two uninfected rhesus macaques.

We used widely accepted mucosal ILC and NK cell surface markers to identify these cell types in the lamina propria of SIV-infected and uninfected colons of rhesus macaques (17). Specifically, we identified ILCs and NKs as CD45^+^ lineage-negative (Lin^−^) (CD3^−^, CD20^−^, CD11c^−^, CD34^−^, CD68^−^, CD123^−^, CD303^−^, FceRI^−^) viable single cells (Fig. 1A and B). CD56^+^ NKs were defined as CD56^+^ CD127^−^ Lin^−^, ILC1s as CD127^+^ CD117^−^ Lin^−^, and ILC3s as CD127^+^ CD117^+^ Lin^−^ (Fig. 1A and B). We first looked at the presence of ILC2s, which also express CD127 and CD117 (17). Unlike ILC3s, ILC2s express interleukin-1 receptor-like 1 (i.e., ST2) (41). Very few CD127^+^ CD117^+^ ST2^+^ lymphocytes (means ± standard deviations [SD], 5.38% ± 1.5%) were identified in SIV-infected colon (Fig. 2A), while ∼20% of CD4^+^ T cells from the same colon were ST2^+^ (Fig. 2B). Moreover, we only detected ∼8% of IL-13 (an ILC2 cytokine [42]) expressing CD117^+^ CD127^+^ Lin^−^ CD45^+^ cells in SIV-infected or uninfected colons. In addition, we found no IL-13-expressing CD117^−^ CD127^+^ Lin^−^ CD45^+^ cells in SIV-infected or uninfected colons (Fig. 2C). Thus, ILC2s were not further investigated in our study.

**FIG 3:**
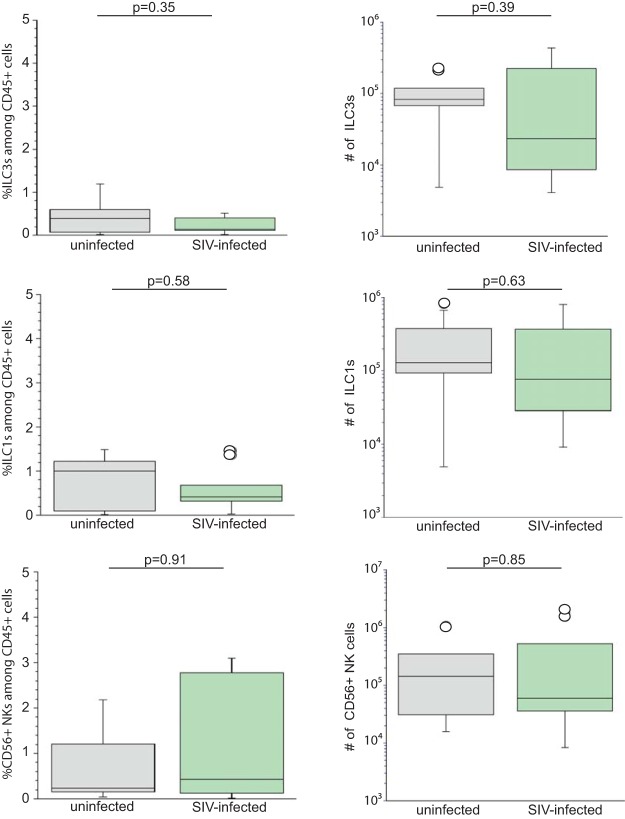
SIV infection does not alter the proportion or number of ILCs and NKs within the lamina propria of the colon. (A, C, and E) Percentage of ILC3s (A), ILC1s (C), and CD56^+^ NKs (E) among the viable CD45^+^ cells from the colons of ten uninfected (gray bars) and ten infected (green bars) rhesus macaques. (B, D, and F) The number of ILC3s (B), ILC1s (D), and NK cells (F) in the colon of ten uninfected (gray bars) and ten infected (green bars) rhesus macaques are shown. The horizontal line in boxes indicates the median, bars are the upper and lower quartiles, and vertical lines indicate variability outside the upper and lower quartiles. Individual points are outliers. The *P* values for statistical significance were determined using Mann-Whitney U test. The *P* value threshold for significance was *P* < 0.05.

When we determined the frequency of ILC3s (Fig. 3A), ILC1s (Fig. 3C), and CD56^+^ NKs, defined phenotypically (Fig. 3E) within the CD45^+^ cells of the colon, we found no difference between infected and uninfected colon.

Just like the frequency of ILC3s, ILC1s, and CD56^+^ NKs within the lamina propria of the colon, the absolute numbers of ILC3s, ILC1s, and CD56^+^ NKs among the CD45^+^ cells of the colon were not changed as a result of SIV infection (Fig. 3B, D, and F).

### SIV infection does not alter the frequency and total number of ILCs and NKs constitutively expressing IL-22.

In most studies of the impact of SIV on ILCs in the GI tract, ILC3s were essentially defined as CD3^−^ cells that have the potential to express IL-22 (32–34). Because ILC3s and ILC1s all can express IL-22 (37–39), these prior studies may not have considered that all possible IL-22-expressing cells were ILC1s and NKs. Here, we will use widely accepted surface markers for identifying ILC3s, ILC1s, and NKs to determine the contribution of each innate lymphocyte subset in IL-22 expression in the lamina propria of the colon.

**FIG 4:**
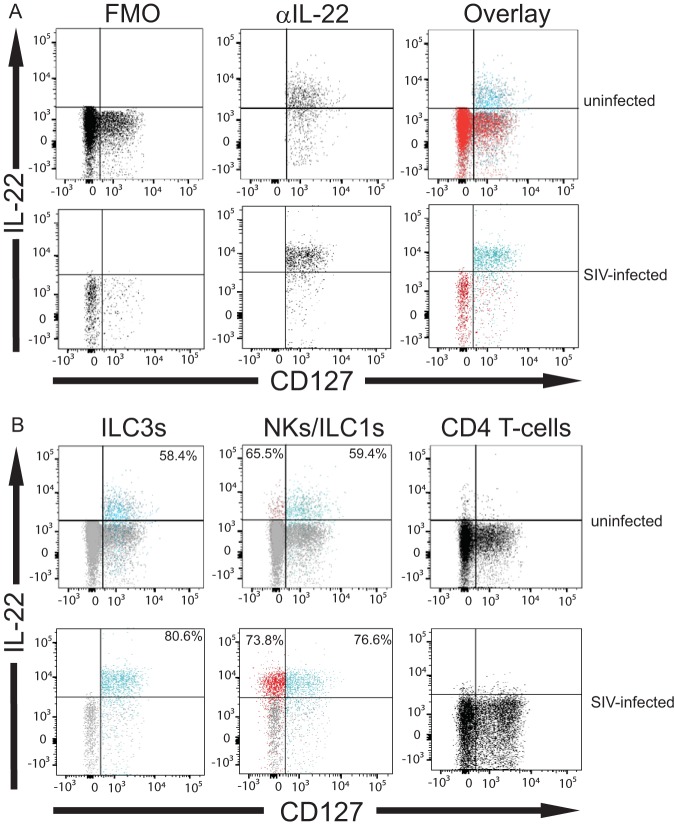

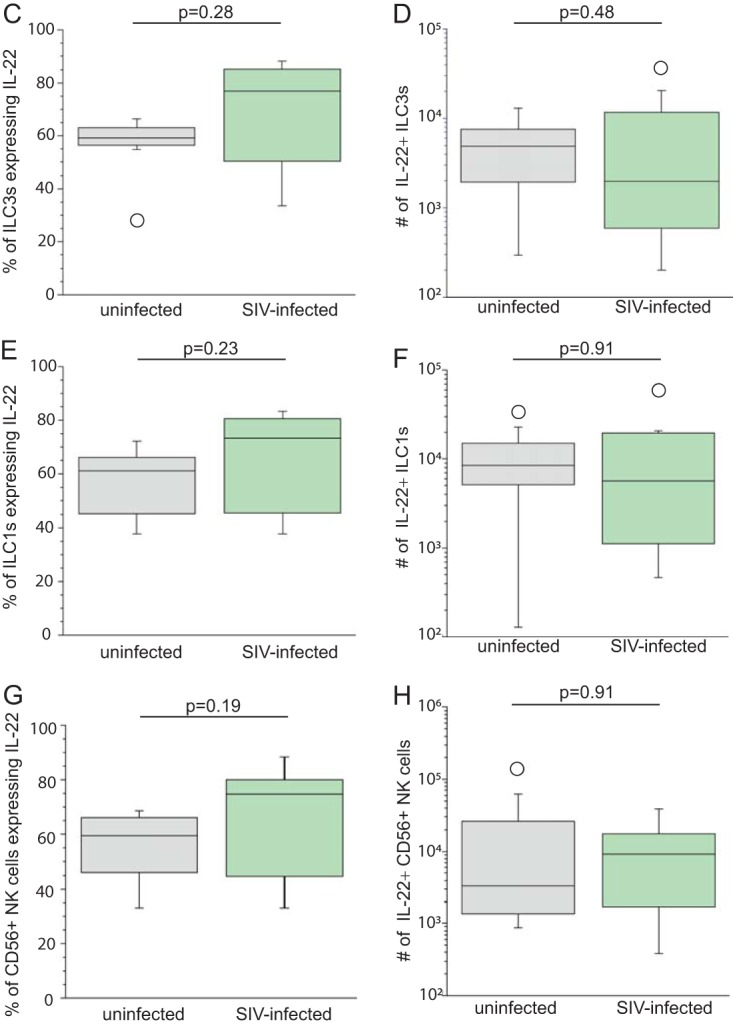
SIV infection is associated with no change in the frequency and number ILC3s, ILC1s, and NKs expressing interleukin-22 within the lamina propria of the colon. (A) Example of ILC3 expression of IL-22 (blue) overlaid with IL-22 FMO (red) from uninfected and SIV-infected colons. (B) Example of ILC3s (blue), ILC1s (blue), NKs (red), and T cells (black) expressing IL-22 from uninfected and SIV-infected colons. Gates were determined using IL-22 FMO controls (gray). Numbers in the upper right corners are frequency of ILC3s or ILC1s expressing IL-22. Numbers in upper left corners are frequency of CD56^+^ NKs (red) expressing IL-22. CD4^+^ T cells in both uninfected and SIV-infected colons do not express IL-22, so no numbers are displayed. (C) Percentage of ILC3s producing IL-22 in the colons of ten uninfected (gray boxes) and infected (green boxes) rhesus macaques. (D) Number of ILC3s expressing IL-22 in the colons of ten uninfected (gray boxes) and ten infected (green boxes) rhesus macaques. (E) Percentage of ILC1s producing IL-22 from uninfected and infected rhesus macaques. (F) Number of ILC1s producing IL-22. (G) Percentage of CD56^+^ NK cells producing IL-22 from uninfected and infected rhesus macaques. (H) Number of CD56^+^ NK cells producing IL-22. Horizontal lines in boxes indicate medians, boxes are the upper and lower quartiles, and vertical lines indicate variability outside the upper and lower quartiles. Individual points are outliers. The *P* values for statistical significance were determined using Mann-Whitney U test. The *P* value threshold for significance was *P* < 0.05.

We directly evaluated the constitutive expression of IL-22 (without exogenous stimulation) by colonic ILC3s *ex vivo* (*n* = 10 SIV-infected rhesus macaques, *n* = 10 uninfected rhesus macaques) (examples are shown in Fig. 4A and B). On average, ∼50% of ILC3s (means ± SD, 55.22% ± 11.98%) from uninfected animals expressed IL-22 (Fig. 4C). However, unlike previous studies looking at the colons of infected animals (32, 33, 36), we observed no statistically significant difference of ILC3s producing IL-22 in the infected colon (means ± SD, 68.60% ± 18.21%) (Fig. 4C) relative to the uninfected colon (*P = *0.28).

Similarly, when evaluating ILC1s for their expression of IL-22, we found no statistically significant change in the frequency of IL-22^+^ ILC1s within SIV-infected colon (means ± SD, 63.07% ± 15.28%) relative to ILC1s from the uninfected colon (means ± SD, 53.05% ± 18.05%; *P = *0.23) (Fig. 4E). Similarly, the percentage of IL-22^+^ colonic NKs in infected tissue (means ± SD, 62.79% ± 19.27%) did not differ much from that observed for the frequency of IL-22^+^ colonic NKs from the uninfected tissue (*P = *0.19) (Fig. 4G). We did not observe IL-22 expression by T cells, indicating that the staining is not due to nonspecific binding of anti-IL-22 antibody to lymphocytes (Fig. 4B). When we analyzed IL-22-producing innate lymphocytes of infected colon relative to that of the uninfected colon, there was no change in the number of ILC3s, ILC1s, or NKs producing IL-22 in the colon tissue (Fig. 4D, F, and H).

### SIV infection does not alter the frequency or number of IL-17-producing ILC3s, ILC1s, and NKs.

**FIG 5:**
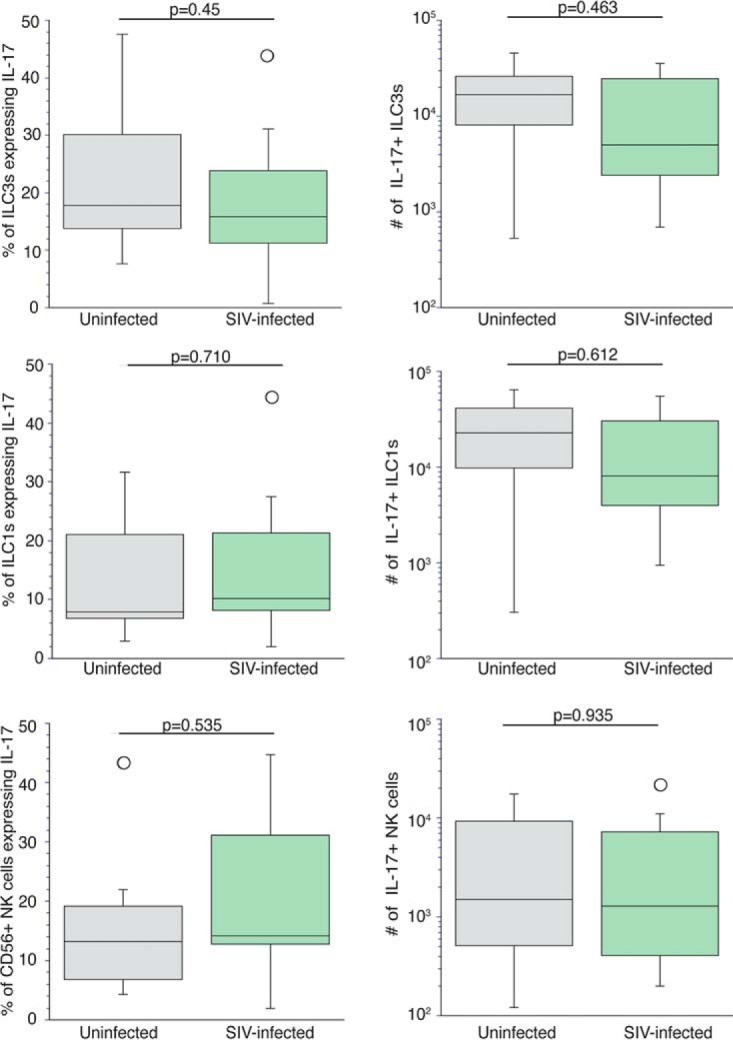
SIV infection is not associated with increased frequency and number of ILC3s, ILC1s, or NKs expressing interleukin-17 within the lamina propria of the colon. (A) Percentage of ILC3s producing IL-17 from the colons of six uninfected (gray boxes) and seven infected (green boxes) rhesus macaques. (C and E) Percentage of ILC1s (C) and NKs (E) producing IL-17 from six uninfected and seven infected rhesus macaques. (B, D, and F) Number of ILC3s (B), ILC1s (D), and NKs (F) producing IL-17. Horizontal lines in boxes indicate the medians, boxes are the upper and lower quartiles, and vertical lines indicate variability outside the upper and lower quartiles. Individual points are outliers. The *P* values for statistical significance were determined using Mann-Whitney U test. The *P* value threshold for significance was *P* < 0.05.

IL-22 and IL-17 may be expressed together within ILCs (43). Therefore, we next determined whether SIV infection is associated with an alteration in both the frequency and number of ILCs and NKs producing IL-17. Unlike IL-22, we found a slightly lower percentage of colonic ILC3s producing IL-17 among the seven infected rhesus macaques (means ± SD, 17.19% ± 14.27%) compared to the six uninfected monkeys in the control group (means ± SD, 23.03% ± 17.80%) (Fig. 5A). However, this difference did not reach statistical significance. Moreover, we found no difference in the number of ILC3s producing IL-17 from the colon (Fig. 5B). In addition to ILC3s, SIV infection did not alter the percentage or number of IL-17^+^ ILC1s (Fig. 5C and D) and NKs (Fig. 5E and F) within the colon.

### Impact that SIV infection has on the frequency and number of IFN-γ-producing ILCs and NKs in the colon.

**FIG 6:**
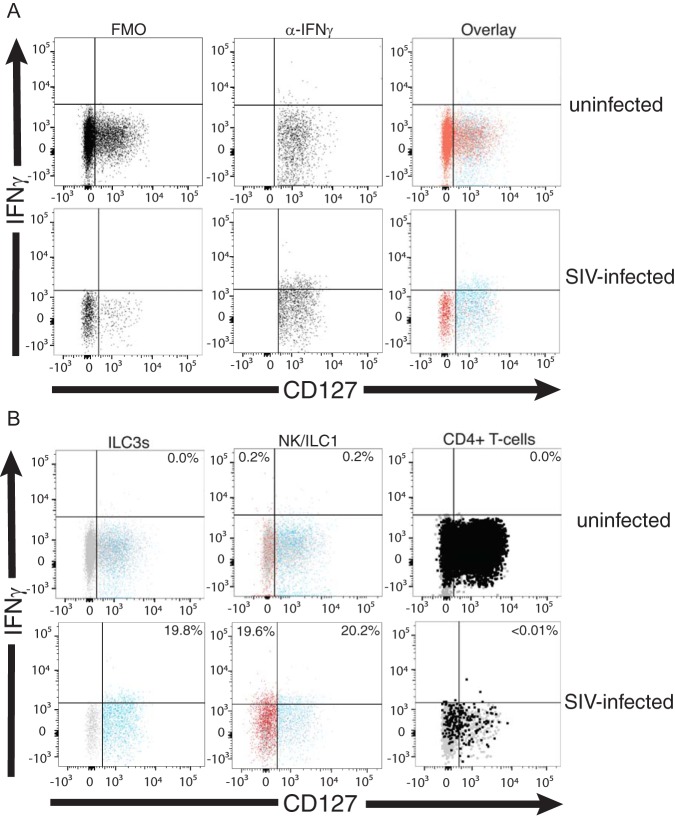

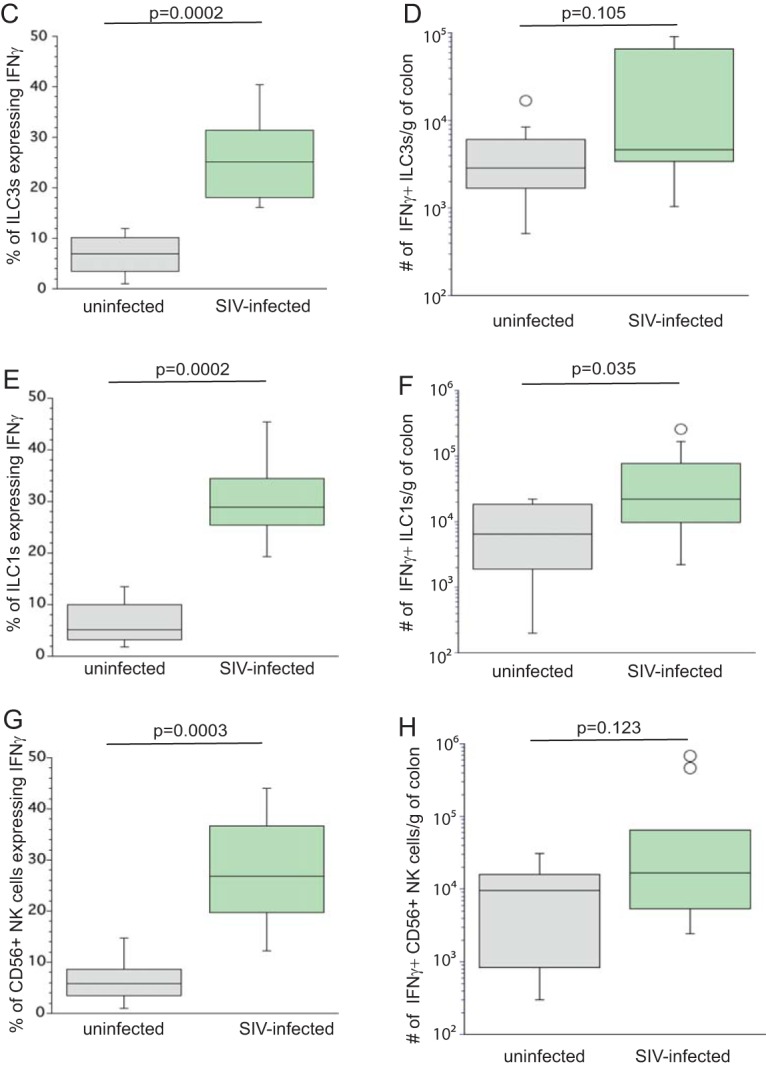
Percentage and number of ILC3s, ILC1s, and NKs expressing IFN-γ in uninfected and SIV-infected colons. (A) FMOs are in the left column, and anti-IFN-γ-stained cells are in the center column. In the right column is ILC3 expression of IFN-γ (blue) overlaid with IFN-γ FMO (red) from uninfected and SIV-infected colons. (B) Example of ILC3s (blue), ILC1s (blue), NKs (red), and T cells (black) expressing IFN-γ from uninfected and SIV-infected colons. Gates were determined using IFN-γ FMO controls (gray). Numbers in upper right corners are frequency of ILC3s or ILC1s expressing IFN-γ. Numbers in upper left corners are frequency of CD56^+^ NKs (red) expressing IFN-γ. CD4^+^ T cells in both uninfected and SIV-infected colons do not express IFN-γ, so no numbers are displayed. (C) Percentage of ILC3s producing IFN-γ in the colons of ten uninfected (gray boxes) and ten infected (green boxes) rhesus macaques. (D) Number of ILC3s expressing IFN-γ in the colons of ten uninfected (gray boxes) and ten infected (green boxes) rhesus macaques. (E) Percentage of ILC1s producing IFN-γ from ten uninfected and ten infected rhesus macaques. (F) Number of ILC1s producing IFN-γ in the colon. (G) Percentage of CD56^+^ NK cells producing IFN-γ from ten uninfected and ten infected rhesus macaques. (H) Number of CD56^+^ NK cells producing IFN-γ in the colon. The horizontal lines in boxes indicate the medians, boxes are the upper and lower quartiles, and vertical lines indicate variability outside the upper and lower quartiles. Individual points are outliers. The *P* values for statistical significance were determined using Mann-Whitney U test. The *P* value threshold for significance was *P* < 0.05.

Since there appears to be no difference in the frequency and number of ILCs and NK cells expressing IL-22 and IL-17 when obtained from SIV-infected colons compared to the same cells within uninfected colons, we sought to evaluate the *ex vivo* expression of inflammatory cytokines like IFN-γ among colonic ILCs and NKs isolated from the colons of ten SIV-infected and ten uninfected rhesus macaques. We found that the percentages of ILC3s constitutively producing IFN-γ (examples are shown in Fig. 6A and B) were significantly higher (*P* = 0.0002) in colons of SIV-infected (means ± SD, 27.30% ± 11.06%) rhesus macaques than colons of uninfected (means ± SD, 5.69% ± 4.08%) rhesus macaques (Fig. 6C). Similarly, we found that a low percentage of ILC1s (means ± SD, 5.69% ± 4.21%) and NKs (means ± SD, 5.40% ± 4.36%) from uninfected colons constitutively expressed IFN-γ *ex vivo*. In contrast, a significantly higher frequency of ILC1s (means ± SD, 30.08% ± 7.47%) and NKs (means ± SD, 26.76% ± 10.61%) from infected colons constitutively expressed IFN-γ (*P* = 0.0002 and *P* = 0.0003) (Fig. 6E and G). Indeed, the percentage of ILC3s producing IFN-γ in colons of infected animals was statistically similar to what we found for IFN-γ-producing ILC1s and NKs in infected colons (Fig. 6C, E, and G).

While there is an increased frequency of ILC3s and NKs producing IFN-γ in the colon of SIV-infected rhesus macaques compared to that of uninfected animals, we found no statistical difference in the number of IFN-γ^+^ ILC3s and NKs in the colon of the infected and uninfected animals (Fig. 6D and H). In contrast, we found a statistically significant increase (*P = *0.035) in the number of ILC1s producing IFN-γ in the colons of infected versus uninfected rhesus macaques (Fig. 6F). It should be noted that collectively (i.e., ILC3s, ILC1s, and NK cells), the number of innate lymphocytes (i.e., ILC3s, ILC1s, and NK cells) that express IFN-γ in the infected colons is statistically different (*P* = 0.0232) from that of IFN-γ^+^ innate lymphocytes from uninfected colons (data not shown).

**FIG 7:**
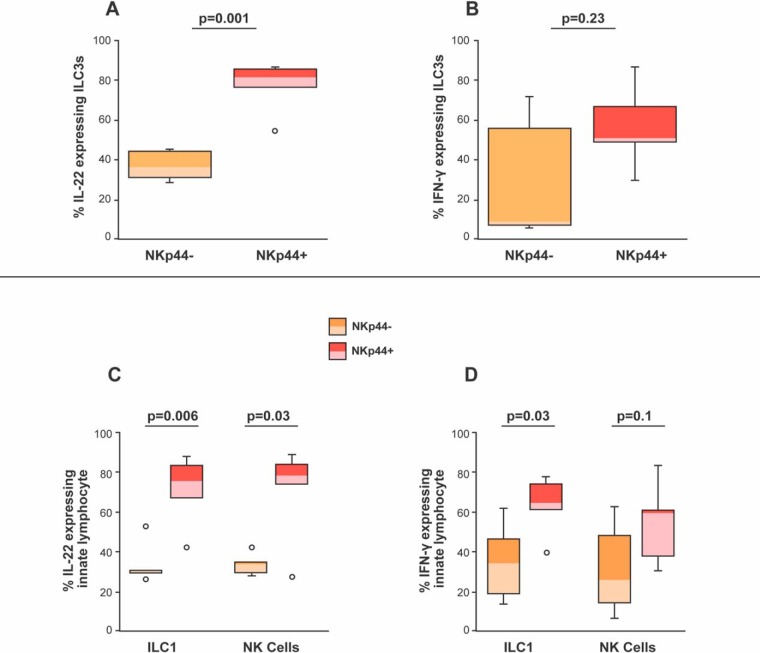
Percentages of NKp44^−^ and NKp44^+^ ILC3s, ILC1s, and NKs expressing IL-22 or IFN-γ in SIV-infected colons. (A) Percentages of NKp44^−^ (orange boxes) and NKp44^+^(red boxes) ILC3s producing IL-22 were determined in the colons of six infected rhesus macaques. (B) Percentages of NKp44^−^ and NKp44^+^ ILC3s producing IFN-γ were determined from the colons of six infected rhesus macaques. (C) Percentages of NKp44^−^ and NKp44^+^ ILC1s and NKs producing IL-22 were determined from the colons of six uninfected and six infected rhesus macaques. (D) Percentages of NKp44^−^ and NKp44^+^ ILC1s and NKs producing IFN-γ were determined from the colons of six uninfected and six infected rhesus macaques. Horizontal lines in boxes indicate medians, boxes are the upper and lower quartiles, and vertical lines indicate variability outside the upper and lower quartiles. Individual points are outliers. The *P* values for statistical significance were determined using Mann-Whitney U test. The *P* value threshold for significance was *P* < 0.05.

We also looked at cells expressing the surface molecule NKp44. Other investigators have reported that IL-22-secreting ILC3s express NKp44 within infected GI tissue (32, 34). Indeed, we found a statistically significant percentage of IL-22-producing, NKp44-expressing ILC3s relative to ILC3s lacking NKp44 in the infected colon (Fig. 7A). NKp44^+^ cells also expressed IFN-γ. However, statistically, there were minimal differences in the frequency of NKp44^+^ and NKp44^−^ ILC3s expressing IFN-γ (Fig. 7B).

We next wanted to determine if the IFN-γ-producing ILC1s also were expressing NKp44. ILC3s are plastic not only functionally but also phenotypically (38, 39). Specifically, ILC3s tend to lose cKIT (i.e., CD117) expression when they are exposed to proinflammatory cytokines (e.g., IL-12), making them look like ILC1 (38, 39). However, despite losing CD117, ILC3s retain their NKp44 when they are exposed to proinflammatory cytokines (38, 39).

Like ILC3s, we found that NKp44 was expressed on ILC1s and NKs and that these cells had a statistically higher expression of IL-22 than the ILC1s and NKs lacking NKp44 (*P = *0.006 and *P = *0.03) (Fig. 7C). While our findings support the notion that NKp44^+^ ILCs are more likely to produce IL-22 than NKp44^−^ ILCs in the context of SIV infection, in contrast to earlier published studies (32), we also found that NKp44^+^ ILC1s were capable of expressing IFN-γ to a statistically more significant degree than NKp44^−^ ILC1s (*P* = 0.03) (Fig. 7D). These findings indicate that IL-22 is not the only cytokine expressed by NKp44-expressing ILCs and that NKp44^+^ ILC1s (i.e., formerly ILC3s) from infected colons are capable of producing IFN-γ.

**FIG 8:**
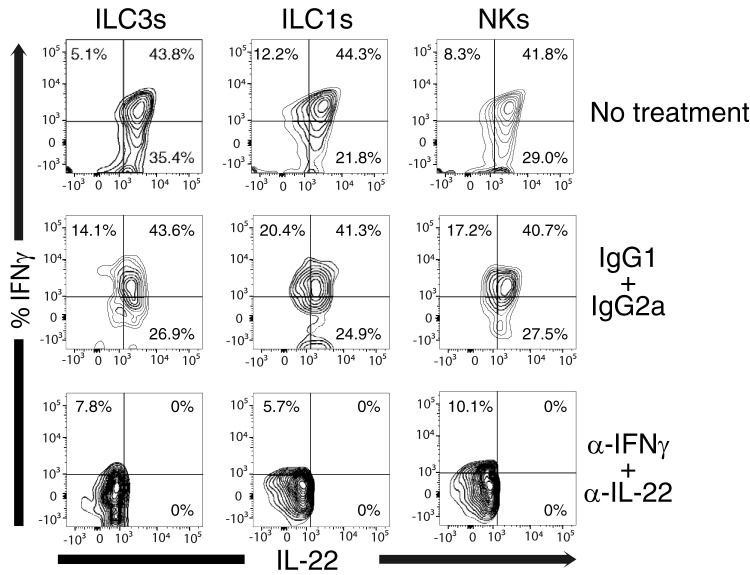
ILCs and NKs coexpress IFN-γ and IL-22 when obtained from the colon of SIV-infected rhesus macaques. Cells from a representative colon were surface stained to identify ILCs and NKs, permeabilized/fixed, and then exposed to unlabeled anti-IFN-γ and IL-22 antibody or IgG1 and IG2a (isotype controls) for 30 min at 4°C at an antibody concentration of four times the amount of fluorochrome-conjugated antibody, which was added in the next step. The cells then were washed twice and stained with fluorochrome-conjugated anti-IL-22 and IFN-γ (same clone as the unlabeled antibodies). Gates were determined using fluorescent minus one (FMO) controls of IFN-γ and IL-22. This is a representative of three separate experiments.

In our study, we found that the anti-IFN-γ antibody explicitly bound to IFN-γ, since the addition of unlabeled anti-IFN-γ prevented fluorochrome-conjugated anti-IFN-γ from staining the cells while unlabeled isotype controls were unable to block fluorochrome-conjugated anti-IFN-γ binding (Fig. 8). This was also true of IL-22, where unlabeled anti-IL-22 blocked the ability of fluorochrome-conjugated anti-IL-22 to bind the cells while unlabeled isotype control antibody had no effect on staining.

**FIG 9:**
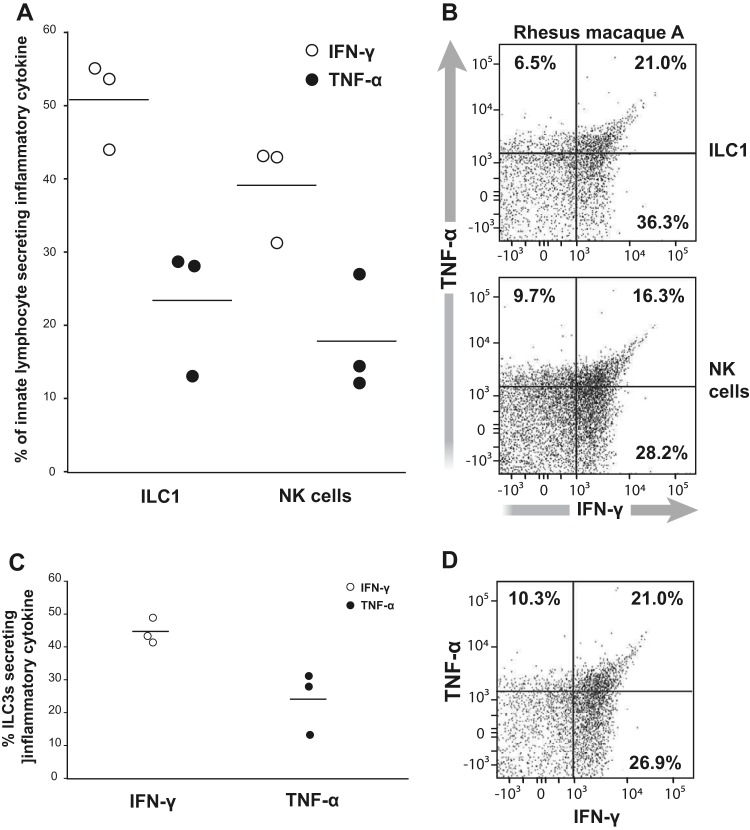
TNF-α and IFN-γ production by ILC3s, ILC1s, and NKs. (A and C) IFN-γ (open circle) or TNF-α (closed circle) from ILC1s and NKs (A) and ILC3s (C) measured from three different SIV-infected colons. Bars in the dot plot represent the means from three IFN-γ- or TNF-α-producing ILC1s or NKs. (B and D) Example of ILC3s, ILC1s, and NKs expressing TNF-α and IFN-γ. Gates are set based on FMOs for TNF-α and IFN-γ staining.

SIV infection is associated with increased epithelial cell leakage (44). TNF-α acts as an inflammatory cytokine that modulates tight-junction proteins on epithelial cells in the presence of IFN-γ (12, 13). We hypothesized that there is a higher number of cells expressing IFN-γ that also express TNF-α. In the infected colon, fewer (typically ∼2-fold) ILCs expressed TNF-α than expressed IFN-γ (Fig. 9A and B). We also uncovered that TNF-α was coexpressed with IFN-γ in the innate lymphocytes (Fig. 9A and C). Like the ILC1s and NKs, we noted the concomitant expression of TNF-α and IFN-γ among the ILC3s (Fig. 9C and D) in infected animals. These studies indicate that ILCs and NKs from the colon of SIV-infected rhesus macaques are capable of producing both IFN-γ and TNF-α.

### ILC3s, ILC1s, and NKs in the colon of rhesus macaques express both T-bet and RORγT.

**FIG 10:**
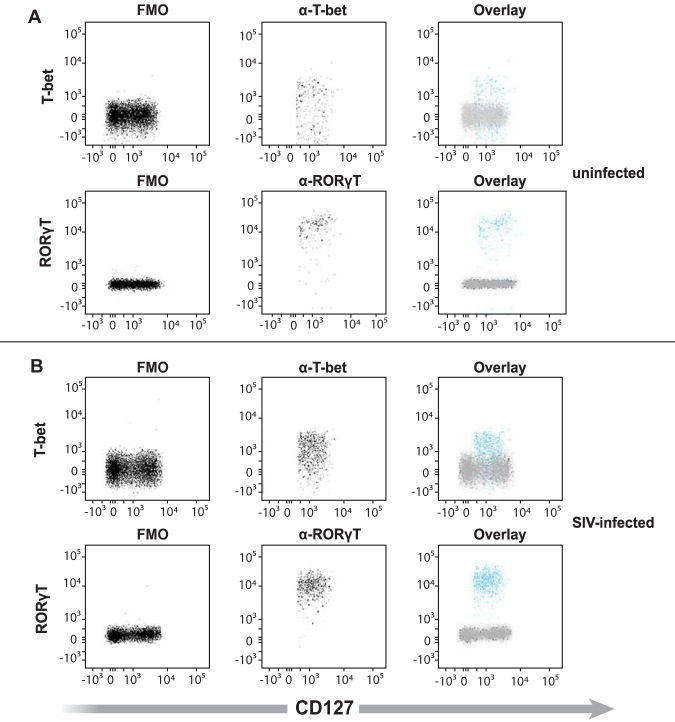

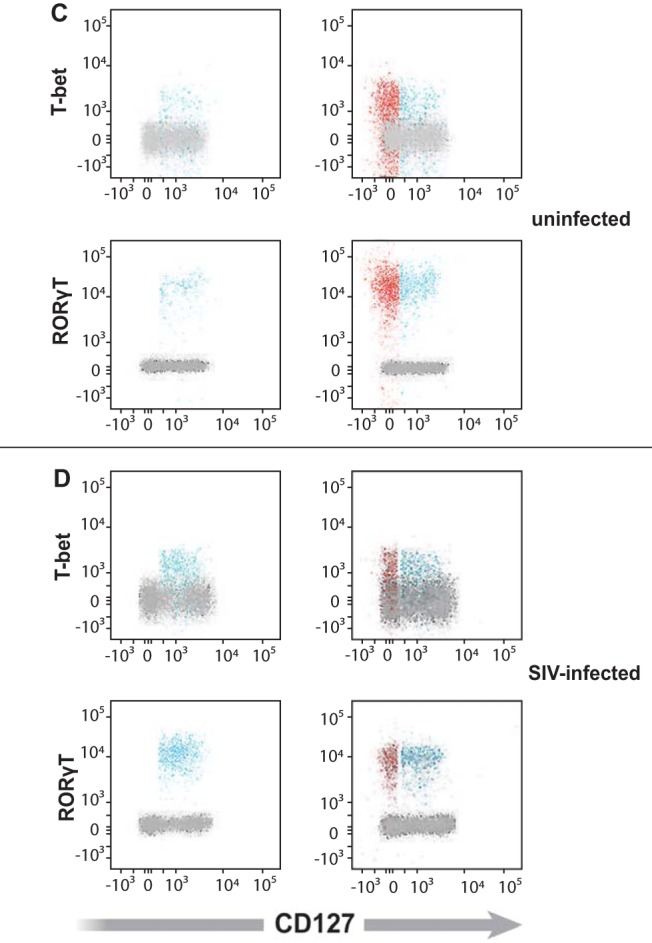
ILC3s, ILC1s, and NKs expressing T-bet and/or ROR-gamma T from the colon of uninfected and SIV-infected rhesus macaques. Example of ILC3s expressing the transcription factors T-bet and ROR-γT (blue) overlaid with FMO (gray) from uninfected (A) and SIV-infected (B) colons. Examples of ILC3s (blue), ILC1s (blue), and NKs (red) expressing T-bet and ROR-γT in uninfected (C) and SIV-infected (D) colons. Gates were determined using FMO controls for T-bet and ROR-γT (gray). This is a representative of four separate experiments.

The expression of IL-22 and IFN-γ by ILCs and NKs likely corresponds to the expression of the transcription factors RORγT and T-bet, respectively (17). We observed ILCs and NKs coexpressing IL-22 and IFN-γ (Fig. 8). Thus, we next determined whether there was a difference in the extent to which ILCs from infected versus colonic ILC subtypes coexpressed RORγT and T-bet in both uninfected (*n* = 4) and SIV-infected (*n* = 4) colons. We found that T-bet and RORγT were expressed in ILC3s, ILC1s, and NKs of both uninfected and infected colons (Fig. 10).

**FIG 11:**
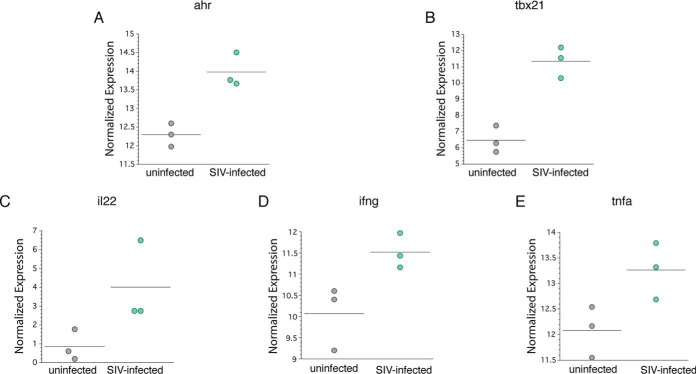
Gene expression by purified NKs from the colons of SIV-infected and uninfected rhesus macaques. Purified NKs from the lamina propria of three uninfected and three SIV-infected rhesus macaques were evaluated for the expression of the genes *ahr* (A), *tbx21* (B), *il22* (C), *ifng* (D), and *tnfa* (E), as determined by real-time reverse transcription PCR. Relative expression of specific genes was normalized based on the global expression of all detected transcripts (Table 2). The horizontal lines indicates the means.

We next determined, in purified NKs from three SIV-infected and three uninfected colons, the expression of transcripts, using quantitative reverse transcription-PCR (RT-PCR) to determine the relative level of aryl hydrocarbon receptor (*ahr*), a key transcription factor for IL-22 expression (Fig. 11A), and *tbx21*, which is essential for IFN-γ expression (Fig. 11B). Many different approaches for quantitation of gene expression have been adapted over the years, depending on the experiment performed: (i) the total RNA or rRNA approach, (ii) the housekeeping gene approach for single-gene analyses, and (iii) the globalization approach for multigene analyses in particular. Given that different normalization approaches significantly changed the *P* values and fold changes of a large number of genes in reference 45, we used global gene expression for analyzing individual outcomes of target gene expression.

Our findings suggest that NKs in the colon of rhesus macaques can express IL-22 and IFN-γ based on the expression of both *ahr* (Fig. 11A) and *tbx21* (Fig. 11B). We found relatively elevated expression of *IL-22*, (Fig. 11C), *ifng* (Fig. 11D), and *tnfa* (Fig. 11E) within the purified NK cell from three SIV-infected compared to three uninfected colons.

### Increased presence of IFN-γ/TNF-α-expressing ILCs and NKs in the colon of SIV-infected rhesus macaques is coupled with TJ protein in serum and altered serum levels of microbial translocation markers.

**FIG 12:**
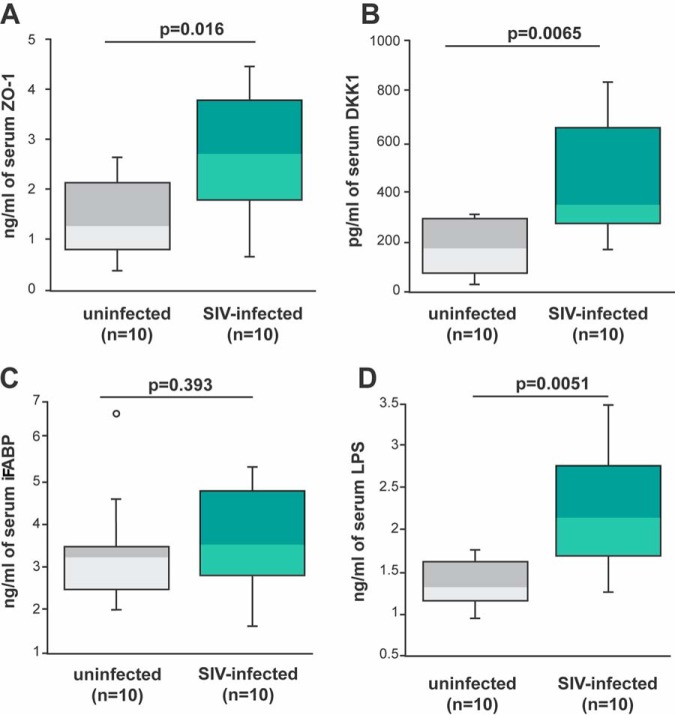
Serum/plasma markers of microbial translocation in SIV-infected and uninfected rhesus macaques. (A) Concentration of zonula occludens-1 (ZO-1) in the serum of the ten uninfected and ten SIV-infected animals used in the studies described in Fig. 4 and 6 were determined by ELISA. (B) Serum concentration of Dickkopf-related protein 1 (DKK1) was determined by ELISA. (C) Serum concentration of intestinal fatty-acid binding protein (iFABP) was determined by ELISA. (D) Concentration of lipopolysaccharide (LPS) in serum taken from the ten uninfected (gray bars) and ten SIV-infected (green bars) rhesus macaques used in the studies described in Fig. 4 and 6 was determined by *Limulus* amoebocyte lysate assay. Horizontal lines in boxes indicate the medians, boxes are the upper and lower quartiles, and vertical lines indicate variability outside the upper and lower quartiles. Individual points are outliers. The *P* values for statistical significance were determined using Mann-Whitney U test. The *P* value threshold for significance was *P* < 0.05.

Increased loss of tight junctions between colonic epithelial cells has been hypothesized to occur as a consequence of exposure of the epithelial cells to IFN-γ and TNF-α (11–13). We determined whether these TJ proteins were found at different levels in the serum/plasma of SIV-infected and uninfected macaques. We found an increased presence of TJ protein ZO-1 in the plasma of infected rhesus macaques compared to that of uninfected animals (*P = *0.016) (Fig. 12A). We also note an increase in DKK-1 in the serum of infected rhesus macaques compared to that of uninfected animals (*P = *0.0065) (Fig. 12B). DKK-1 is a known inhibitor of Wnt signaling, which is critical for both intestinal stem cell development and e-cadherin-dependent tight junctions in the GI tract (46–49).

One other possibility is that IFN-γ and TNF-α lead to higher shedding of colonic epithelial cells (49). iFABP is a 12-kDa cytoplasmic protein located in intestinal enterocytes involved in the uptake and transport of lipids (50, 51). iFABP is commonly released in serum when epithelial cells in the GI tract undergo cell death (52). We found no difference in iFABP in the circulation of infected and uninfected animals (*P = *0.393) (Fig. 12C). These findings support that IFN-γ and TNF-α produced by ILCs in infected rhesus macaques weaken tight junctions within the colon rather than leading to the loss of colon cells.

One of the hallmarks of SIV/HIV infection is the translocation of microbial products (3). It has been hypothesized that these microbial products originate from the intestinal lumen and enter into the mucosa by microbial translocation (44). Eventually, the microbial products wind up in circulation (53). As expected (3), we found an elevated level of lipopolysaccharide (LPS) within the serum of SIV-infected rhesus macaques compared to that of uninfected animals (*P = *0.0051) (Fig. 12D).

## DISCUSSION

In our study, we revealed a feature of unaltered frequency and the number of innate immune cells, based on phenotype, within SIV-infected rhesus macaque colon tissue. Specifically, we found that the number of ILC3s, ILC1s, and NKs within the colon tissue was not changed as a result of infection (Fig. 3). Moreover, there were no changes in the frequency of ILC3s, ILC1s, and NK cells among the CD45^+^ cells in the colon (Fig. 3). With one exception (35), previous studies identified ILC3s within the GI tract of SIV-infected rhesus macaques (32–34, 36) using different approaches that are inconsistent with more recent efforts to identify these cells based on the coexpression of CD127 and CD117 following the exclusion of other cells that may express these surface molecules (17). Moreover, prior approaches assumed that group 3 ILCs were the exclusive innate lymphoid cell type able to express IL-22 (15). Our findings now show that other ILCs, based on phenotype, within the colon are capable of producing IL-22 constitutively (Fig. 4). Our study sheds new light on the biology of rhesus macaque colonic NK and ILCs. We also find that IFN-γ, traditionally thought to be an ILC1/NK-exclusive cytokine (15), is expressed by a significant proportion of ILC3s during SIV infection (Fig. 6). Several studies have claimed that rather than being static in the type of cytokines expressed, ILCs and NK cells are plastic both functionally and phenotypically. Evidence for this claim stems from studies illustrating that ILC and NK clones secrete specific cytokines depending on the polarizing cytokines they are exposed to (e.g., IL-23/IL-1β and IL-12/IL-18) (38, 39). Thus, colonic ILC1s and NKs could express IL-22 in uninfected colons, and, similarly, ILC3s could express IFN-γ in the setting of SIV infection.

As noted in our studies, we report both the frequency and number of ILCs within colon tissue. However, flow cytometry of digested tissue is not optimal as a means to provide an accurate count of cells within the tissue. The best means of determining the number of cells within tissues at this time is microscopy (54). We are hindered in enumerating ILCs and NK cells by microscopy by the lack of a specific marker to identify an ILC, and only four fluorochromes may be used. The identification of ILCs relies on the elimination of leukocyte subpopulations, which share markers associated with ILCs (i.e., CD127 and CD117). Unfortunately, CD56, used to identify NK cells within tissue, also stains neurons in colon tissue (55). Moreover, we and others (56) have found that CD56 is also found on ILC1s and ILC3s in the colon. NKp44 and NKG2A were used as a means of differentiating rhesus macaque mucosal ILCs from NK cells in one study (32). However, we found that NKp44^+^ and NKp44^−^ ILCs and NK cells can produce both IFN-γ and IL-22 (Fig. 7). NKG2A-positive ILCs also express IL-22 and IFN-γ to the same degree as NKG2A-negative ILCs (data not shown). Thus, NKp44 and NKG2A may not be useful in identifying and enumerating ILCs and NK cells within colons of SIV-infected animals.

Previous studies of ILCs in SIV-infected rhesus macaques (32–36) utilized mitogens to stimulate the cells to secrete cytokines *in vitro*. While this type of stimulation provides information about the potential of the cells to express cytokines, it does not directly reflect constitutive cytokine expression occurring *in vivo*. We did not stimulate the cells in our studies. All rhesus macaque colons used in our study were from fresh tissue taken within minutes of necropsy and processed immediately, followed by freezing in liquid nitrogen (LN_2_). In addition, we did not utilize Golgi-blocking additives (e.g., monensin and brefeldin), as most *in vitro* studies of intracellular cytokines have used these, which may account for some of the differences observed.

There is concern over the low level of anti-IFN-γ or TNF-α detected by the flow cytometer in our studies, and what little fluorescence that is detected may be autofluorescence or nonspecific binding of fluorescent antibody. Unfortunately, we have to use antibodies to human IFN-γ and TNF-α, which may not be optimal for staining for rhesus antigens without *in vitro* stimulation. The detection of autofluorescence would have been observed in our fluorescent minus one (FMO) controls and gated out. Autofluorescence is primarily a biological factor in cells that fluoresce naturally when the biomolecules are excited by the flow cytometer laser. Nonspecific binding of fluorochrome-conjugated antibodies was addressed in our studies shown in Fig. 8. The unlabeled isotype control did not alter the percentage of cytokine-expressing ILCs, but the unlabeled anti-cytokine antibodies altered the staining of the fluorescent cytokine antibody. Thus, we were not dealing with autofluorescence or nonspecific binding of fluorescent antibody in our study when we detected IFN-γ or TNF-α.

The findings of the constitutive expression of cytokines are not limited to our study. Using immunohistochemical techniques of fixed/frozen GI tissue, other investigators found innate lymphocytes capable of expressing cytokines without the need for *in vitro* stimulation. In one such study, a decreased number of ILC3s from colon biopsy specimens of HIV-infected patients express IL-17 and IL-22 relative to that of uninfected donors (27). ILC3s from uninfected rhesus macaques also have been shown to constitutively express IL-17 in the jejunum *in situ* (36). It should be noted that in both studies, ILC3s are not identified based on accepted phenotype markers. We found that the constitutive expression of IL-17 and IL-22 (Fig. 4 and 5) is not limited to the ILC3 subset but is also expressed by ILC1s and NKs in the uninfected colon. Although our studies described here were primarily in colon, we have found that similar frequencies of ILCs express IL-22 or IL-17 in both uninfected and infected ileum (data not shown).

Mucosal NKs, like ILC1s, typically secrete IFN-γ and TNF-α (15). Surprisingly, we found that colonic NKs from both infected and uninfected animals also expressed IL-22 (Fig. 4). This finding was shown not only using flow cytometry but also by RT-PCR of purified colonic NKs (Fig. 11). Consequently, whereas NKs from the colon may be characterized phenotypically based on their expression of CD56 and lack of expression of CD127 and CD117, functionally they are very similar to ILC1s. ILC1s could originate from ILC3s that have lost c-Kit (38, 39) while retaining the ability to secrete IL-22. There are no reports to date indicating that mucosal tissue ILC1s convert to NKs (57). The other possibility is that colon-specific NKs differ from NKs in the lymphoid tissues and blood and instead mirror the function of ILCs found in GI tissue (58).

In the present study, we evaluated only CD56^+^ NKs. In the context of HIV infection, blood-derived NKs lose CD56 but retain CD16 (59, 60). When we evaluated the subpopulations of CD56 and/or CD16 expressing NKs in the colon of five SIV-infected animals, we observed a similar frequency of CD56^+^ CD16^−^ and CD56^+^ CD16^+^ NKs producing IFN-γ and TNF-α relative to that of CD56^−^ CD16^+^ NKs (data not shown). However, the role that the CD56^−^ CD16^+^ population of NKs plays in HIV pathogenesis in the GI tract requires further evaluation beyond the scope of our current study.

In the colon of SIV-infected rhesus macaques, we noted that ILCs and NKs expressed IL-22 and IFN-γ simultaneously (Fig. 8). We observed concomitant increases in fluorescence in the anti-IL-22-stained cells with increases in fluorescence with anti-IFN-γ-stained cells. This is also observed for the IFN-γ and TNF-α staining seen in Fig. 9. The pattern noted is not due to improper compensation, since the experimental staining groups utilized in our studies included FMO controls in every experiment. The fluorophores we utilized in our FMO control were the same, and the brightness and voltage settings were similar to the ones used in our experimental samples. Moreover, we clearly collected enough events in controls to be statistically significant. As shown in Fig. 8, the pattern of staining went away when unlabeled anti-cytokine antibodies were added to the cells before adding the fluorochrome-conjugated antibody. In contrast, preincubation with unlabeled isotype control did not remove the pattern of staining observed in our untreated controls.

We also noted that colonic ILC3s, ILC1s, and NKs coexpressed T-bet and RORγT (Fig. 10), the transcription factors that are necessary for IFN-γ and IL-22 expression, respectively, regardless of infection status. We found that purified NKs from the colon express both *tbx21* and *ahr* by RT-PCR (Fig. 11). Although we used *ahr* instead of *rorg* in the RT-PCR, both transcription factors expressed by the genes are necessary for IL-22 expression (15). Interestingly, we found that ILCs and NKs from the colon of uninfected rhesus macaques also coexpressed both T-bet and RORγT (or *tbx21* and *ahr* for purified NKs). This finding indicates that ILCs can secrete both cytokines and are poised to secrete either cytokine (e.g., IFN-γ or IL-22) depending on the mucosal environment (e.g., IL-18 or IL-23). However, despite the expression of both sets of transcription factors, NKs and ILCs from uninfected rhesus macaques minimally expressed IL-22 and IFN-γ. Studies in mouse models have previously demonstrated that GI tract CCR6-NKp46^+^ ILCs coexpressing T-bet and RORγT produce IL-22 in the presence of IL-23 and produce IFN-γ when stimulated with IL-12, but not IL-23, *in vitro* (61). Furthermore, microbiota within the colon of mice (in the presence of IL-23) is necessary for the expression of T-bet by RORγT expressing NKp46^+^ CCR6^−^ ILCs (61). The data from this previous study and our own indicate that ILCs and NKs from the gut of SIV-infected rhesus macaques generate both IFN-γ and IL-22 if the correct stimuli are present. Additional evidence of ILCs coexpressing ILC3- and ILC1-type cytokines in humans was from a very recent study by investigators utilizing RNA velocity analysis that identified an intermediate ILC3-ILC1 cluster, which had strong directionality toward ILC1s (62). These ILC3-ILC1 transition cells were found in the lamina propria of the human ileum, suggesting that ILC3-to-ILC1 plasticity is common to mucosal tissues and could coexpress both IFN-γ and IL-22, which we have shown, in our studies, is possible. Together, the findings in our studies indicate that in the context of HIV/SIV, IL-22-expressing ILCs are present. IL-22 is critical in maintaining colon epithelial cell health via such mechanisms as the induction of antimicrobial peptide gene expression by deep crypt secretory cells (63), increasing the fucosylation of epithelial cells (24), and enhancement of proliferation by transit-amplifying cells (64).

We noted in our study that the increased presence of IFN-γ/TNF-α-producing ILCs in the GI tract of SIV-infected rhesus macaques was connected with increases in serum LPS compared to that of uninfected animals (Fig. 12). These findings indicate that IFN-γ/TNF-α in the GI tract likely leads to microbial translocation as a consequence of two possible mechanisms. First, IFN-γ/TNF-α can lead to the downregulation of TJ proteins (11–13). Second, the increase in TNF-α can lead to an increase in the loss of epithelial cells in the colon (49, 65). Based on the data presented here and the increased presence of transit-amplifying cell replication with increased shedding of epithelial cells during HIV infection (66), we conclude that the loss of TJs between epithelial cells during HIV/SIV infection is more likely the basis of microbial product translocation. Further studies are needed to determine whether the loss of the epithelial barrier during SIV/HIV infection is due to the paracellular movement of microbial products from the lumen to the mucosal tissue or increased loss of epithelial cells during SIV/HIV infection.

## MATERIALS AND METHODS

### Ethics statement.

The tissues were provided by the Nonhuman Primate Biological Materials Distribution Core of the Wisconsin National Primate Research Center (University of Wisconsin in Madison). Tissues from this core have been harvested ante- or postmortem from animals assigned to the University of Wisconsin at Madison IACUC-approved research protocols. Because we acquired discarded postmortem tissue, specific protocol numbers are not provided.

### Animals and virus.

A total of 30 Indian-origin rhesus macaques (Macaca mulatta) were used in this study. Fifteen (eight males, seven females; average age, 7.4 years; range, 1.8 to 14.9 years) were infected with SIV_Mac239_. Uninfected animals in our study were age- and gender-matched with infected animals. Animals infected with SIV that had no overt sign of clinical disease were sacrificed between 6 and 24 months following infection using humane approaches. Serum was collected, and colons were necropsied immediately and tissue placed on ice until being picked up from the facility and processed in a biosafety level III facility at the AIDS Vaccine Research Laboratory at the University of Wisconsin (<30 min after necropsy).

### Cell isolation and processing.

Colon tissue sections (average area, 3 cm by 8 cm) were processed immediately for single-cell suspensions. Fatty tissue and feces were removed and tissue cleaned and washed in phosphate-buffered saline (PBS) before being weighed. For uninfected colons, the mean weight ± standard deviations was 10.9 ± 2.8 g, and infected colon was 10.6 ± 2.4 g. Colon sections first were manually dissected into 2.0-cm sections and then treated with 0.33 M dithiothreitol (DTT) (Sigma, St. Louis, MO) in Hanks balanced salt solution (HBSS) and Primocin (Fisher Scientific, Pittsburgh, PA) to remove the mucus. Five-millimeter strips of tissues were further incubated for two 50-min cycles (37°C with constant shaking at 225 rpm) with 2 mM ethylenediaminetetraacetic acid (EDTA) (Gibco) in 0.1% bovine serum albumin (BSA; Fisher) in HBSS to remove intraepithelial leukocytes. Finally, tissue was minced into 1-mm pieces and treated for two 60-min incubations (37°C with shaking at 225 rpm) with 10 mg/ml collagenase II from Clostridium histolyticum (Gibco) in RPMI 1640 medium containing 2% BSA and Primocin to isolate lamina propria leukocytes. In all steps, cell suspensions were filtered through a 100-μm mesh. After counting, we moved the cells into recovery cell culture freezing medium (Gibco) and froze the contents in cryovials (Corning) at a rate of 1°C per minute in a –80°C freezer overnight before being transferred to liquid nitrogen for cryopreservation.

### Flow cytometry.

Cryovials containing frozen cell suspensions were immediately thawed following removal from liquid nitrogen, washed twice in phosphate-buffered saline (PBS) (Gibco), and counted. No Golgi-blocking additives were used (e.g., monensin and brefeldin). At least 4 × 10^6^ cells were stained in test tubes (Fisher), while staining control tubes contained 10^6^ cells. Cells were first stained with a 1:1,000 dilution of Aqua Dead staining dye (Invitrogen) for 20 min at room temperature in PBS. Cells then were washed with PBS twice to remove the residual dye. For the second staining, cells were incubated in Becton, Dickinson (BD; San Jose, CA) brilliant violet staining buffer and stained with the appropriate concentrations of surface antibodies for 20 min at 4°C (a complete list of antibodies is in Table 1). Fluorescent minus one (FMO) staining control tubes were set up with all antibodies used in the experiment except the one antibody being measured. FMO controls were set up for each antibody tested using the same sample as the experimental group each time. Cells then were washed twice in PBS with 0.01% sodium azide (Sigma). For intracellular staining, cells next were incubated with either BD Cytofix/Cytoperm buffer for cytoplasmic staining or eBioscience Foxp3/transcription factor staining buffer (Fisher) (applied according to the manufacturer’s instructions) to fix and permeabilize cells. Fixed and permeabilized cells then were stained with antibodies to either cytokines or transcription factors.

**TABLE 1:** Antibodies utilized for flow cytometry, blocking, and sorting

Ab	Vendor	Clone	Fluorochrome^*a*^
CD3	BDIS	SP34-2	BV421
CD20	BDIS	L27	BV421
CD45	BDIS	D058-1283	AF700
CD56	BDIS	NCAM 16.2	BV785
CD68	BDIS	Y1/82A	BV421
CD127	Beckman Coulter	R34.34	PE-Cy7
CD11c	BioLegend	3.9	BV421
CD123	BioLegend	6H6	BV421
CD34	BioLegend	581	BV421
CD303	BioLegend	201A	BV421
CD117	BioLegend	104D2	PerCP-Cy5.5
IFN-gamma	BioLegend	4S.B3	BV711
CRTH2	BioLegend	BM16	FITC
NKp44	Miltenyi	2.29	FITC
RORYT	Affymetrix	AFK-JS9	PE
TBET	BioLegend	4B10	BV711
IL-22	Affymetrix	IL22-JOP	APC
NKG2A	Beckman Coulter	HP-3B1	FITC
IL-17	Affymetrix	eBio64CAP17	APC-CY7/FITC
TNF-Alpha	BioLegend	MAb11	FITC

a PE, phycoerythrin; PerCP, peridinin chlorophyll protein; FITC, fluorescein isothiocyanate; APC, allophycocyanin.

In experiments testing nonspecific binding of fluorochrome-conjugated antibodies to IFN-γ and IL-22, fixed and permeabilized cells were treated with unlabeled anti-IFN-γ and IL-22 or isotype control antibodies (30 min at 4°C) before adding fluorochrome-conjugated anti-IFN-γ and anti-IL-22 antibodies (clones of fluorochrome-conjugated antibodies are the same as the unlabeled antibodies).

Multicolor flow cytometry was used to detect ILCs in our study. Stained cells and their controls were collected, and data were processed on a BD LSRFortessa. Data were analyzed using FlowJo software, version 10.15 (FlowJo LLC). The threshold for detection for each cell type was the following: NKs, >1,000 events for both SIV-infected and uninfected rhesus macaques; ILC1s, >1,000 in SIV-infected rhesus macaques and >750 in uninfected rhesus macaques; ILC3s, >500 in SIV-infected rhesus macaques and >1,000 in uninfected rhesus macaques.

For our study, we identified ILCs and NKs in the colon of SIV-infected rhesus macaques using widely accepted markers that were used to identify ILCS and NKs in human mucosal tissues (17). Using FMO staining controls, we first gated samples on CD45^+^ cells and then determined the frequencies of ILC and NKs among lineage-negative (Lin^−^) (CD3^−^, CD20^−^, CD11c^−^, CD34^−^, CD68^−^, CD123^−^, CD303^−^, FceR^−^) viable single cells (Fig. 1). It should be noted that CD68 is a marker that identifies monocytes as well as macrophages (67). All ILCs express CD127. In our studies, we identified NKs as CD56^+^ CD127^−^ Lin^−^. ILC1s were identified as CD127^+^ CD117^−^ Lin^−^. ILC2s and ILC3s express both CD127 and CD117 (17). Unlike ILC3s, ILC2s express ST2 (42).

The number of cells of tissue was determined using the following specific formula: number of ILCs or NK cells from the colon = (total number of cells recovered from the colon)(percent CD45^+^ cells)(percent single cells)(percent viable cells)(percent Lin^−^ cells)(percent ILCs or NK cells).

### Real-time RT-PCR and normalization.

Quantitative reverse transcription PCR (qRT-PCR)-based expression profiling was performed on the BioMark HD system using Dynamic Array integrated fluidic circuit (Fluidigm, South San Francisco, CA). Assays for the Delta Gene project were designed in D3 Assay Design Studio (Fluidigm). Total cellular RNA was purified and DNase treated using an miRNeasy Micro kit and RNase-free DNase set (Qiagen, Valencia, CA) according to the manufacturer’s protocol. RNA samples then were analyzed according to the BioMark RT-PCR protocol of the real-time PCR analysis user’s guide (PN 68000088 N1) by Fluidigm. In brief, RNA samples were reverse transcribed and underwent TSA (target-specific preamplification) using a multiplex of assays. cDNA was preamplified for 18 PCR cycles. Preamplified cDNA was diluted 1:5 and used to set up RT-PCRs with each assay. Samples were analyzed using four technical replicates.

For normalization in our studies, we based mRNA expression of target genes on quantitative RT-PCR of 13 transcripts (Table 2) that encoded proteins of various functions, including, but not limited to, cytokines, transcription factors, cytokine receptors, and phenotypic markers. Results from real-time RT-PCR-targeted genes were converted to normalized gene expression values. Gene expression was normalized based on the global expression of all detected transcripts, allowing us to examine differences in the expression of multiple genes, as described in reference 45.

**TABLE 2:** Primers used in RT-PCR

Target	Primer sequence	
	Forward	Reverse
aHR	CAGCGCCAACATCACCTAC	TCAGCCGGGATTGGCTTTA
IFN-γ	GCAGGTGATCCAGATGTAGCA	TTTCTGTCACTCTCCTCTTTCCA
TNF-α	TCCAACCATGTGCTCCTCAC	GATGGCAGAGAGGAGGTTGAC
EOMES	CGGCAAAGCCGACAATAACA	TCTCATCCAGTGGGAACCAGTA
IL-12RB1	CCAGGAACCGGACAGAGAAA	TGTCTCCCAGAGGAGGCTTATA
IL-12RB2	CACCAAGCATTTGCGTTGCTA	GGGCCGCTAGGAAAACAGATA
IL-17a	TGACTCCTGGGAAGACCTCA	GGATTGCTATTCCTGCCTTCAC
IL-18R1	CCCTTGACTCTTTGGGTGCTTA	GGTTCCCCTTCAACCACAGTA
IL-18 RAP x2 variant	ACGGTCAGAGCTGTTGTTCA	GCTCTCGACAGGATCCAGAATA
IL-1RAP	CCAAAGGCGAAGTTGCCAAA	CCAAAACCACAAGCCAGTTCC
IL-22	GTCCAAAAGCTGAAGGACAC	ACAGCAAATCCAGTTCTCCA
IL-23R	CCATCTCCACAGGACACCTTA	ACAGCAAAGACGATCATTCCC
NCR2	CCCCACACCCACTGCTAC	TGCCCTGCCACACTTTGAA
T-box21	AACCACCTGTTGTGGTCCAA	AGCCACCGTAAATGACAGGAA

### ELISAs.

ELISAs for zonula occludens (ZO-1), Dickkopf-related protein-1 (DKK-1), and intestinal fatty acid binding protein (iFABP) were purchased from MyBioSource (San Diego, CA) and were used according to the manufacturer’s instructions. In brief, sera from all animals were stored in a freezer at –80°C and thawed immediately before use. The serum was added to the empty wells of ELISA plates containing standard reagents, and blank wells for controls were incubated with detection antibody and washed, detection was reagent added, and then plates were read at 420 nm on a Cytation 3 imaging reader (Biotek, Winooski, VT). Concentrations of samples were calculated according to the manufacturer’s (MyBioSource) formula.

### LPS in serum measurements.

Serum LPS was measured, from both uninfected and SIV-infected rhesus macaques, using the *Limulus* amoebocyte lysate (LAL) chromogenic endotoxin quantitation kit (Pierce Biotechnology, Rockford, IL) according to the manufacturer’s instructions. In brief, sera alongside standards were incubated with LAL reagent at 37°C for 10 min, and then the substrate was added for an additional 6 min before the stop solution was added. Samples and controls then were read out at 405 nm on a Cytation 3 imaging reader (Biotek). LPS concentration was calculated using the manufacturer’s formula (Pierce Biotechnology) from the obtained values created by the standard curve.
